# Supplementary material for: Systematic Review of Pain in Clinical Practice Guidelines for Management of COPD: A Case for Including Chronic Pain?
Source: Healthcare (Basel). 2019 Jan 22;7(1):15. doi: 10.3390/healthcare7010015 (PMC6473434; doi:10.3390/healthcare7010015)
Supplement: Supplementary file 1 [file healthcare-07-00015-s001.pdf]

Table S1: The search strategy conducted in OVID Medline.

Search number	Search term and limits
1	COPD.mp
2	Chronic obstructive pulmonary disease.mp
3	Pulmonary emphysema.mp
4	exp*Pulmonary Disease, Chronic Obstructive/
5	1 OR 2 OR 3 OR 4
6	Guideline.m_titl
7	Consensus.m_titl
8	Position statement.m_titl
9	Guidance.m_titl
10	Standard.m_titl
11	6 OR 7 OR 8 OR 9 OR 10
**12 (Final search)**	**5 AND 11**

Table S2: The clinical practice guidelines for the management of chronic obstructive pulmonary disease (COPD) included in this review.

Clinical practice guidelines title	Year	Reference
**Updated guidelines**		
Global strategy for the diagnosis, management, and prevention of chronic obstructive pulmonary disease: 2019 report	2019	**UD** GOLD: International [1]
The COPD-X Plan: Australian and New Zealand Guidelines for the management of Chronic Obstructive Pulmonary Disease 2018	2018	**UD** Australia and NZ [2]
Guideline for the Diagnosis and Treatment of COPD Patients Issued by the German Respiratory Society and the German Atemwegsliga in Cooperation with the Austrian Society of Pneumology	2018	**UD** Germany [3]
Diagnosis, Prevention and Treatment of Stable COPD and Acute Exacerbations of COPD: The Swiss Recommendations 2018	2018	**UD** Swiss [4]
Revised (2018) COPD Clinical Practice Guideline of the Korean Academy of Tuberculosis and Respiratory Disease: A Summary	2018	**UD** Korea [5]
National guidelines for care for asthma and COPD—Support for management	2018	**UD** Sweden [6]
Chronic Obstructive Pulmonary Disease (COPD): Diagnosis and Management	2017	**UD** BC: Canada [7]
Joint statement for the diagnosis, management, and prevention of chronic obstructive pulmonary disease for Gulf Cooperation Council countries and Middle East-North Africa region, 2017.	2017	**UD:** N Africa/ME [8]
Chronic Obstructive Pulmonary Disease	2017	**UD** Michigan: USA [9]
Chronic obstructive pulmonary disease MOH clinical practice guidelines	2017	**New** Singapore [10]
**Clinical guidelines identified in the original search (published 2006–2016)**		
Global strategy for the diagnosis, management, and prevention of chronic obstructive pulmonary disease: updated 2016	2016	GOLD: International [11]
The COPD-X Plan: Australian and New Zealand Guidelines for the management of Chronic Obstructive Pulmonary Disease 2016	2016	Australia and NZ [12]

Health Care Guideline: Diagnosis and Management of Chronic Obstructive Pulmonary Disease (COPD)	2016	ICSI: USA [13]
National guidelines for care for asthma and COPD—Support for management	2015	Sweden [14]
NHG-Standaard COPD (Derde herziening) (NHG Standard COPD (Third Revision))	2015	NHG: Netherlands [15]
The Saudi Guidelines for the Diagnosis and Management of COPD	2014	Saudi Arabia [16]
Guidelines for Diagnosis and Management of Chronic Obstructive Pulmonary Disease	2014	India [17]
The clinical and Integrated management of COPD. An official document of AIMAR (Interdisciplinary Association for Research in Lung Disease), AIPO (Italian Association of Hospital Pulmonologists), SIMER (Italian Society of Respiratory Medicine), SIMG (Italian Society of General Medicine)	2014	Italy [18]
VA/DoD clinical practice guideline for the management of chronic obstructive pulmonary disease	2014	VA/DoD: USA [19]
Russian Respiratory Society Federal guidelines on diagnosis and treatment of chronic obstructive pulmonary disease	2015	Russia [20]
Keuhkoahtaumatauti (COPD) ukaine	2014	Finland [21]
Zalecenia Polskiego Towarzystwa Chorób Płuc dotyczące rozpoznawania i leczenia przewlekłej obturacyjnej choroby płuc (Polish Respiratory Society Guidelines for Chronic Obstructive Pulmonary Disease)	2014	Poland [22]
Guide du parcours de soins Bronchopneumopathie chronique obstructive (Guide term care Chronic Obstructive Pulmonary Disease)	2014	HAS: France [23]
Kroni̇k obstrükti̇f akci̇ğer hastaliği (koah) koruma, tani ve tedavi̇ raporu 2014 (Chronic obstructive lung disease (COPD) protection, diagnosis, and treatment report 2014)	2014	Turkey [24]
COPD 2014	2014	Korea [25]
Guía Latinoamericana de EPOC—2014 (ALAT–COPD Guideline: an evidence-based assessment)	2014	Latin America [26]
Guía Espa˜nola de la EPOC (GesEPOC). Tratamiento farmacológico de la EPOC estable (Spanish Guide for COPD (GesEPOC). Pharmacological treatment of stable COPD)	2014	Spain [27,28]

Chronic Obstructive Pulmonary Disease: Official diagnosis and treatment guidelines of the Czech Pneumological and Phthisiological Society; a novel phenotypic approach to COPD with patient-oriented care	2013	Czech Republic [29]
Diagnosis and Management of Chronic Obstructive Pulmonary Disease: The Swiss Guidelines	2013	Switzerland [30]
慢性阻塞性肺疾病诊治指南(2013 年修订版) (Guidelines for the Diagnosis and Treatment of Chronic Obstructive Pulmonary Disease (Revised 2013))	2013	China [31]
Guía Clínica Enfermedad Pulmonar Obstructiva Crónica EPOC (Clinical Guide Chronic Obstructive Pulmonary Disease COPD)	2013	Chile [32]
Хронічне Обструктивне Захворювання Легені Адаптована Клінічна Настанова, Заснована На Доказах (Chronic Obstructive Pulmonary Disease Adapted Clinical Guidelines Based on Evidence)	2013	Ukraine [33]
Diagnóstico e Tratamento da Doença Pulmonar Obstrutiva Crónica (Diagnosis and Treatment of Chronic Obstructive Pulmonary Disease)	2013	Portugal [34]
(COPD national academic policy and guidelines for prevention, diagnosis, and care)	2012	Norway [35]
Chronic Obstructive Pulmonary Disease	2012	Michigan: USA [36]
PCO Prise en Charge De la Broncho-Pneumopathie Chronique Obstructive Guide pratique à l'usage du praticien (Chronic Obstructive Pulmonary Practice Guide for the Practitioner)	2012	Algeria [14]
Danske KOL-Guideline (Danish COPD guideline)	2012	Denmark [37]
Recomendaciones para la prevención, diagnóstico y tratamiento de la epoc en la Argentina (Recommendations for the prevention, diagnosis, and treatment of COPD in Argentina)	2012	Argentina [38]
Diagnosis and Management of Stable Chronic Obstructive Pulmonary Disease: A Clinical Practice Guideline Update from the American College of Physicians, American College of Chest Physicians, American Thoracic Society and the European Respiratory Society	2011	USA/Europe [39]
Guideline for the management of Chronic obstructive pulmonary disease—2011 update	2011	South Africa [40]
Chronic obstructive pulmonary disease (COPD)	2011	BC: Canada [41]
Guía de práctica clínica Diagnóstico y tratamiento de la enfermedad pulmonar obstructiva crónica (Clinical Practice Guideline Diagnosis and Treatment of Chronic Obstructive Pulmonary Disease)	2011	Mexico [42]

Chronic obstructive pulmonary disease: Management of chronic obstructive pulmonary disease in adults in primary and secondary care	2010	United Kingdom [43]
Richtlijn Diagnostiek en behandeling van COPD (Guideline for the diagnosis and treatment of COPD)	2010	NVALT: Netherlands [44]
Recommandation pour la Pratique Clinique Prise en charge de la BPCO Mise à jour 2009 (Recommendation for Clinical Practice Management of COPD Updated 2009)	2010	SPLF: France [45]
Management of Chronic Obstructive Pulmonary Disease	2009	Malaysia [46]
Clinical Practice Guidelines in the Diagnosis and Management of Chronic Obstructive Pulmonary Disease (COPD) in the Philippines	2009	Philippines [47]
Canadian Thoracic Society recommendations for management of chronic obstructive pulmonary disease—2007 update ***Plus update:*** Canadian Thoracic Society recommendations for the management of chronic obstructive pulmonary disease—2008 update—highlights for primary care	2007/08	CTS: Canada [48,49]
Leitlinie der Deutschen Atemwegsliga und der Deutschen Gesellschaft für Pneumologie und Beatmungsmedizin zur Diagnostik und Therapie von Patienten mit chronisch obstruktiver Bronchitis und Lungenemphysem (COPD)(Guidelines for the Diagnosis and Therapy of COPD Issued by Deutsche Atemwegsliga and Deutsche Gesellschaft für Pneumologie und Beatmungsmedizin)	2007	Germany [50]
International Primary Care Respiratory Group (IPCRG) Guidelines: Management of Chronic Obstructive Pulmonary Disease (COPD)	2006	IPCRG: International [51]
Chronic Obstructive Pulmonary Disease Consensus Recommendations for Early Diagnosis and treatment	2006	Texas: USA [52]

Table S3: The mentions of "pain" in the clinical practice guidelines for the management of COPD.

Guideline	Mentions of "pain" *n*	Mention of "pain" verbatim	Context of "pain" mention
**CPGs updated since 2016: new "pain" mentions**			
**UD** Australia and NZ **2018** [2]	1	In section Manage Exacerbations, "Based on the high prevalence of pulmonary embolism, this diagnosis should be considered in patients presenting with an exacerbation of COPD when signs of an infection are absent and **chest pain** and cardiac failure are present." p. 128	Physician visit to assess for differential diagnosis (chest pain)
**UD** German **2018** [3]	5	In section Methylxanthine, "Significant undesirable effects include nausea, vomiting, abdominal pain, sleep disturbances, muscle cramps, hypocalcaemia, and in particular atrial and ventricular, sometimes life-threatening cardiac arrhythmia, as well as cerebral seizures. These are observed especially in elevated theophylline serum levels, while side effects such as headache, drowsiness, nausea, **chest pain** are also found in normal serum levels." p. 267	Drug adverse effects (methylxanthines)
		In section Nicotine Replacement Therapy, "All nicotine replacement therapies are well tolerated for individual application-specific side effects (patches: skin irritation, patch allergies, chewing gum and tablet: irritation of the oral mucosa, tongue, throat, esophagus and stomach in the form of **burning pain**, ulcerations and hiccups; mucous membrane in the form of **burning pain**, atrophy)." p. 275	Drug adverse effects (Nicotine replacement therapies)
		In section Phosphodiesterase-4 Inhibitors, "The most common adverse effects of Roflumilast are nausea, poor appetite, **abdominal pain**, diarrhea, sleep disorders and headache." p. 271	Drug adverse effect (phosphodiesterase-4 inhibitors)
	Re-worded	In section Palliative Care, "Shortness of breath, tiredness, anxiety, depression and **pain** of varying organ localization are the main symptoms in patients in the last year before their death." p. 279	Symptom of COPD
**UD** Korea **2018** [5]	1	In section Phosphodiesterase-4 Inhibitors "The most frequent adverse effects are nausea, reduced appetite, weight loss, **abdominal pain**, diarrhea, sleep disturbances, and headache." p. 265	Drug adverse effects (phosphodiesterase-4 inhibitors)

**UD** BC: Canada **2017** [7]	1	In Appendix A: Prescription Medication Table for COPD1-4,<br>"Roflumilast, Therapeutic Considerations**:** Potential adverse effects: diarrhea, weight loss (average of 2 kg), nausea, headache, **abdominal pain**." p. 5	Drug adverse effects (phosphodiesterase-4 inhibitors)
**New** Singapore **2017** [10]	1	In section Roflumilast,<br>"Roflumilast is associated with fairly severe gastrointestinal side effects (nausea, diarrhoea and **abdominal pain**), headache, and weight loss10, and psychiatric events such as insomnia, anxiety and depression." p. 36	Drug adverse effects (phosphodiesterase-4 inhibitors)
**CPGs identified in the original search: all mentions of "pain"**			
GOLD: International [11] **2016**	1	In section Phosphodiesterase-4 Inhibitors Adverse Effects,<br>"Phosphodiesterase-4 inhibitors have more adverse effects than inhaled medications for COPD. The most frequent adverse effects are nausea, reduced appetite, **abdominal pain**, diarrhea, sleep disturbances, and headache." p. 25	Drug adverse effects (phosphodiesterase-4 inhibitors)
Australia and NZ [12] **2016**	4	In section Oral Bronchodilators, Methylxanthines,<br>"Theophylline has some efficacy in COPD but due to its potential toxicity (the most common adverse reactions being gastric irritation, nausea, vomiting, anorexia, **epigastric pain**, reactivation of peptic ulcer, gastro-oesophageal reflux, haematemesis, tachycardia, palpitations, headache, CNS stimulation, reflex hyperexcitability, insomnia and tremor (MIMS Australia Pty Ltd 2008), inhaled bronchodilators are preferred when available (Global Initiative for Chronic Obstructive Lung Disease (GOLD) 2006)." p. 38	Drug adverse effects (methylxanthines)
		In section Oral Bronchodilators, Phosphodiesterase type-4 Inhibitors,<br>"Drug related adverse effects mainly affect the gastrointestinal system; diarrhoea, **abdominal pain**, nausea and vomiting and weight loss are approximately twice as common in subjects taking PDE-4 inhibitors as in those taking placebo." p. 39	Drug adverse effects (phosphodiesterase-4 inhibitors)
		In section Palliation and End of Life Issues,<br>"The World Health Organisation defines palliative care as an approach that improves the quality of life of patients and their families facing the problem associated with life-threatening illness, through the prevention and relief of suffering by means of early identification and impeccable assessment and treatment **of pain** and other problems, physical, psychosocial and spiritual." p. 71	Palliative care for management and assessment (non-specific)

		In section Confirm Exacerbation and Categorise Severity, Arterial Blood Gas,<br>"The primary reasons for preferring VBG (over ABG) samples cited by the authors were **less pain** and lower risk of bruising." p. 100	Lung function testing indication/contraindication
NHG: Netherlands [15]<br>**2015**	2	In section Diagnosis,<br>"In the case of differential diagnosis between COPD and heart failure, pay attention to cardiovascular risk factors or conditions in the history, such as hypertension, a myocardial infarction or angina pectoris, and ask for topical complaints, such as palpitations or **chest pain** that fits angina pectoris."	Physician visit to assess for differential diagnosis (chest pain)
		In section Evaluation,<br>"In differential diagnostics, the following conditions are particularly important:<br>* Lung carcinoma; their suspicion may be based on symptoms (haemoptysis, altered cough pattern, **chest pain**, weight reduction) or on a chance finding on a chest X-ray…."	Physician visit to assess for differential diagnosis (chest pain)
Saudi Arabia [16]<br>**2014**	1	In section Phosphodiesterase-4 Inhibitors,<br>"The most common side effects are nausea**, abdominal pain**, diarrhea, reduced appetite, headache, and sleep disturbance. Most of these adverse effects improve over time. Mild weight reduction was also reported." p. 62	Drug adverse effect (phosphodiesterase-4 inhibitors)
India [17]<br>**2014**	2	In section: What are Symptoms of COPD?<br>**"Chest Pain** and Haemoptysis: These are not the usual symptoms of COPD. Their presence is often a pointer to an **alternative diagnosis** e.g., lung malignancy, PTB etc.)." p. 18	Physician visit to assess for differential diagnosis (chest pain)
		In section What are the Surgical Treatments that can be Offered for the Treatment of COPD?,<br>"Bullectomy: Bullectomy. The presence of large bullae is one of the common findings in patients with COPD. However, extensive data on the natural history of bullae is not available. Generally, enlargement of these bullae occurs, with complications like dyspnoea, haemoptysis, **chest pain** and pneumothorax. Some the accepted indications for bullectomy are the presence of single large bullae compressing the remaining lung, breathlessness due the bulla, haemoptysis, and reduction in the FEV1 to <50%." p. 41	Symptom of COPD (from bullae)
Italy [18]<br>**2014**	1	In section Palliative and End of Life Care in COPD,	Palliative care for management

		"Palliative care should be integrated within the treatment plan for patients with COPD [9–11] and be initiated when symptoms such as dyspnea, **pain**, depression, anxiety and constipation are not completely controlled by standard pharmacological treatment. The term palliation encompasses interventions aimed at preventing and relieving patient suffering through symptom control, so as to stabilize or improve quality of life." p. 10	
VA/DoD: USA [19] **2014**	3	"One observational study of 242 patients found 10% of patients admitted with COPD exacerbation actually met standard criteria for myocardial infarction (**chest pain** combined with elevated troponin and/or electrocardiogram changes). Therefore, it is important to exclude a myocardial infarction in patients with COPD who present with symptoms and signs suggestive of an exacerbation." p. 25	Physician visit to assess for differential diagnosis (chest pain)
		Following recommendation: We suggest against offering roflumilast in patients with confirmed, stable COPD in primary care without consultation with a pulmonologist. (Weak Against) "In general, adverse events were more common in patients receiving roflumilast compared to placebo. Gastrointestinal events such as diarrhea, nausea, vomiting, dyspepsia, and **abdominal pain** were observed more frequently in patients treated with roflumilast than placebo." p. 33	Drug adverse effects (phosphodiesterase-4 inhibitors)
		In Table D2 Information for Pharmacologic Agents for COPD, by Drug Class, Beta 2-agonists, "May cause palpitations, **chest pain**, rapid heart rate, increased blood pressure, tremor, nervousness." p. 80	Drugs adverse effects (Beta-2 agonists)
Finland [21] **2014**	3	In section Palliative Care, "The most common symptoms of advanced COPD are (% of patients) [41,241,242] • shortness of breath (about 90–95%) • cough (about 50–80%) • **pain (about 30–70%)** • fatigue syndrome (about 70–80%) • depression (about 35–70%) (pain listed as a common symptom of advanced COPD—pain experienced in 30–70% of patients)." p. 25	Symptom of COPD
		In section Palliative Care,	Palliative care for management—with opioids

		"**Pain** should be treated according to management guidelines. Opioids should be used if necessary. The risk of respiratory failure is small, as long as opioids are administered to the extent that symptoms **(pain** or dyspnoea) are alleviated [245,247]." p. 25	
Poland [22] **2014**	2	In section Surgical Treatment of Emphysema,<br>"Emphysema can be removed to control breathlessness, haemoptysis, infection, **chest pain** or to expand the compressed segment." p. 249	Surgical intervention for management of pain
		In section Phosphodiesterase-4 Inhibitor,<br>"Phosphodiesterase inhibitors 4 have more adverse reactions than inhaled medicines for the treatment of COPD. The most common are nausea and diarrhoea, decreased appetite, **abdominal pain** and headaches and sleep disorders." p. 246	Drug adverse effects (phosphodiesterase-4 inhibitors)
HAS: France [23] **2014**	2	As a footnote for the definition of Palliative care,<br>"Palliative care is an active, continuous, evolving, coordinated and practiced by a multidisciplinary team in a global and individualized approach, with the following objectives:<br>• preventing and alleviating **pain** and other symptoms, taking into account psychological, social and spiritual needs, respecting the dignity of the person being cared for;<br>• limit the occurrence of complications, by developing customized prescriptions;<br>• limit the breaks in care by ensuring the good coordination between the different actors of care." p. 28	Palliative care for management
		In Table 8: Smoking cessation drugs,<br>"Nicotinic substitute, Side effects, Systemic: headache, nausea palpitations, insomnia, more rarely abnormal dreams. Oral Forms: hiccups, sore throat, mouth irritations. Transdermal forms: erythema, pruritus or edema, burning at the point of application, **localized muscle pain."** p. 36	Drug adverse effects (Nicotine replacement therapy)
Turkey [24] **2014**	1	In section Roflumilast,<br>"Side effects: **Pain** is one of the other common side effects reported." p. 44	Drug adverse effects (phosphodiesterase-4 inhibitors)
Korea [25] **2014**	6	In section Contraindications, Pulmonary Function Testing,<br>"Some patients who undergo pulmonary function testing require a great deal of physical effort and should not be tested if myocardial infarction has occurred within 17 months. In addition, the following cases cannot obtain accurate test results, so it is a contraindication. - If you cannot cooperate due to chest or abdominal **pain**. - If your mouthpiece is **pain**ful, you have **pain** in	Lung function testing indication/contraindication

		your mouth or face. - If you have stress incontinence. - If you cannot cooperate because of dementia or consciousness.”	
		In section Doxofylinne,<br>“Side effects: Nausea, vomiting, epigastric **pain**, indigestion, heartburn, headache, dizziness, tremor, respiratory distress, tachycardia, Irritability, insomnia.”	Drug adverse effects (doxophylline)
		In section Roflumilast,<br>“Side effects: Muscle weakness, **muscle pain**, **back pain**, fatigue, fatigue….”	Drug adverse effects (phosphodiesterase-4 inhibitors)
Spain [27,28]<br>**2014**	1	In Table 1 Highlights of major comorbidities in COPD patients,<br>“Ischemic heart disease: Diagnostic features: Some common symptoms with COPD: **chest pain** in exacerbations, dyspnea as anginal equivalent.” p. 4 (English 2014 guideline)	Physician visit to assess for differential diagnosis (chest pain)
Czech Republic [29]<br>**2013**	1	In section Palliative Care in COPD,<br>“An important part of palliative care is the administration of opioids (orally, transdermally or parenterally), first justified in discussion with the patient’s family or the patient himself/herself. The main rationale for the use of opioids in this situation is sedation and inhibition of **pain** (e.g. from compressive spinal fractures), and otherwise unmanageable sensation of dyspnoea. Monitored administration of benzodiazepines is also effective at this stage of the disease. Very severe dyspnoea treatment can be supported with inhalation of furosemide and several other non-pharmacological methods—e.g. by cooling the face.” p. 197	Palliative care for management—with opioids
China [31]<br>**2013**	3	In Guideline Preface,<br>“In order to regulate chronic obstructive pulmonary disease, diagnosis and treatment, to ensure the quality of medical care, improve the level of clinical work, and thus effectively reduce the **patient's pain,** improve the quality of life, reduce the death of the disease rate, reduce the burden of disease, 1997 Chinese Medical Association Respiratory Diseases The club has organized relevant experts from China to refer to international experience and combine the actual situation in China has formulated the diagnosis and treatment of chronic obstructive pulmonary disease.”	Non-specific management (goal)
		In section Diagnosis and Differential Diagnosis,<br>“Comprehensive collection of history to assess the diagnosis of chronic obstructive pulmonary disease, the first should be a comprehensive collection of medical history, including .other non-	Physician visit to assess for differential diagnosis (chest pain)

		specific symptoms (wheezing, chest tightness, **chest pain** and morning headache, but also pay attention to the history of smoking (in terms of the year) and occupational, environmental exposure of harmful substances such as history." p. 70	
		In section Phosphodiesterase-4 (PDE-4) Inhibitor<br>"Adverse reactions: the most common are nausea, loss of appetite, **abdominal pain**, diarrhea, sleep disorders and headaches…" p. 73	Drug adverse effects (phosphodiesterase-4 inhibitors)
Chile [32]<br>**2013**	1	In section Recommendations for Diagnosis,<br>"When considering a COPD diagnosis, check for the presence of the following factors: weight loss, stress intolerance, nocturnal arousals, ankle inflammation, occupational hazards, **chest pain**, hemoptysis (11)." p. 16	Physician visit to assess for differential diagnosis (chest pain)
Ukraine [33]<br>**2013**	2	In section Treatment of COPD Exacerbations,<br>"**Chest pain** and fever are uncharacteristic for exacerbation of COPD and should be induced to find another cause of the disease." p. 84	Physician visit to assess for differential diagnosis (chest pain)
		In section COPD Diagnosis,<br>"Patients who are suspected of COPD should also be asked about the following factors:<br>…Is there a **chest pain**? …<br>Note: The last two symptoms are uncharacteristic for COPD and, in their presence, the probability of another disease increases." p. 18	Physician visit to assess for differential diagnosis (chest pain)
Norway [35]<br>**2012**		In section Other Diseases,<br>"Diseases that may give similar symptoms as COPD and which must be ruled out are heart failure, pulmonary artery disease (sarcoidosis, pulmonary fibrosis), sequela after tuberculosis, cystic fibrosis and tumors of the trachea and main bronchi. In exertion-related **chest pain** in addition to shortness of breath, coronary heart disease must be considered, while hemoptysis may include acute respiratory infection, pneumonia, lung cancer or pulmonary embolism." p. 31	Physician visit to assess for differential diagnosis (chest pain)
		In section Laboratory Test,<br>"The test (cardiopulmonary exercise test) can tell whether it is the cardiocirculatory system, lung function, weight, ventilation/hypoxia/hypercapnia, peripheral muscle, **pain** in the musculoskeletal system, motivation/anxiety or irregular breathing patterns, or various combinations of these factors that explain a person's functioning." p. 53	CPET for pain assessment as exercise limitation

		In section Secretion Mobilisation, Techniques for Secretion Mobilisation, Positive Expiratory Pressure (PEP),<br>"For PEP the respiratory tract is kept open during the exhalation and increased pressure with improved air supply to the alveoli can contribute to stretch expansion of atelectatic lung tissue. In practice, PEP is a suitable aid for patients with difficulty to increase ventilation due to low level of function, overweight, pronounced fatigue or **pain** in the thorax." p. 55	Alternative management strategy for patients with pain
		In section Systemic Drug Therapy, Phosphodiesterase Inhibitors (PDE4),<br>"The most common side effects are nausea, **abdominal pain**, diarrhoea, sleep disorders and headaches. The side effects are early treatment is reversible and may decrease with prolonged use." p. 86	Drug adverse effects (phosphodiesterase-4 inhibitors)
		In section Osteoporosis and Fractures,<br>"COPD patients are at increased risk of osteoporosis and fracture. In patients with severe COPD have almost 70% osteoporosis or osteopenia. Hip fractures contributes to an already reduced mobility, while compression breaks, beyond **pain** and functional limitation, also contribute to increasing kyphosis of the thoracic spine, and develop an addition of a spirometric restrictive component and hence more breathing difficulties." p. 106	Complication of COPD
		In section Symptoms and Treatment Measures at Terminal COPD,<br>"The main distressing symptoms in advanced COPD include fatigue, shortness of breath, anorexia, depression, insomnia, **pain** and dry mouth/thirst." p. 117	Symptom of COPD
		In section Opiates,<br>"The main drugs for the relief of heavy breathing are opiates… The dosage of morphine is lower than in **pain**…." p. 118	Non-specific pain management—reference to opioid dose
		In section Appendix, Borg CR10 Scale,<br>"Instruction: You should use this scale to tell how strong your experience or feeling is. It can apply your experience of effort, **pain**, difficulty or anything else." p. 143	Rating scale for pain assessment (non-specific context)
		In section Appendix, Borg CR10 Scale (explanation of the tool),<br>"…The experience of the effort depends primarily on the fatigue in your muscles, whether you feel breathless and eventual **pain."** p. 144	Rating scale for pain assessment (non-specific context)
Algeria [14] **2012**	1	In section Appendix, Six-Minute Walk Test,	Lung function testing indication/contraindication (6MWT)

		"Circumstances of immediate cessation of test (6MWT): **chest pain,** maximal dyspnoea, asphyxia, ramps in the lower limbs, loss of balance, dizziness, pallor, malaise, excess sweating." p. 47	
South Africa [40] **2011**	2	In section Surgery in COPD,<br>"Pre-operative preparation, optimisation of airway function (bronchodilators, steroids, antibiotics and chest physiotherapy), smoking cessation, weight reduction where applicable, delay of elective surgery if chest infection is present, and patient education on deep breathing exercises, coughing, **pain control** and incentive spirometry are recommended." p. 70	Patient education for management (specific to preoperative preparation)
		In section The Six-Minute Walk Test,<br>"The 6MWT **s**hould be discontinued in patients who complain of **chest pain**, intolerable dyspnoea or leg cramps, or appear to be staggering or have an ashen or pale appearance." p. 72	Lung function testing indication/contraindication (6MWT)
United Kingdom [43] **2010**	5	In the definition for Palliative Care,<br>"Palliative care: Care aimed at alleviating symptoms, **pain** and distress, and hence improving quality of life, rather than at curing or slowing progression of a disease or condition. It is often associated with, but is actually not limited to, the end of life." p. 48	Palliative care for management
		In section Symptoms,<br>"Patients in whom a diagnosis of COPD is considered should also be asked about the presence of the following factors: **chest pain**." p. 63	Physician to assess for differential diagnosis (chest pain)
		In section Symptoms of an Exacerbation,<br>**"Chest pain** and fever are uncommon features of COPD exacerbations and should prompt a search for other aetiologies." p. 356	Physician to assess for differential diagnosis (chest pain)
		In section Oxygen Therapy during Exacerbations of COPD,<br>"Radial stabs to obtain blood for arterial blood gas analysis are not more **painful** than arterialised ear lobe gases." p. 384	Lung function testing indication/contraindication (blood gas)
		In section Oxygen Therapy during Exacerbations of COPD<br>"Arterialised ear lobe samples are an alternative way of obtaining arterial blood gases if there is local expertise and may be less **painful** for patients." p. 384	Lung function testing indication/contraindication (blood gas)

NVALT: Netherlands [44] **2010**	10	In section Recommendation,<br>“People with a mucus problem can be stimulated to be active and move in addition to other interventions. Using a simple questionnaire can get an impression of the problems that the patient experience and whether a reference here is useful:<br>Questions concerning sputum retention:<br>- Do you regularly cure (daily) mucus?<br>- Can you easily cough or take a lot of effort?<br>- How long are you coughing up in the morning?<br>- Is it ever black for the eyes when coughing?<br>- Are you very tired or short-tempered after coughing?<br>- Is **coughing painful**?<br>- Is the sputum tough?” p. 106	Questionnaire for assessment (painful cough for mucus clearance)
		In section Quality of Life of People with COPD,<br>“People with mild to moderate COPD treated in general practice score lower on almost all dimensions of quality of life, certainly in comparison with people with asthma. In the patient panel chronic diseases people with COPD were compared with people with other chronic disorders (not asthma) with regard to a number of important aspects of the chronic disease. The differences between the two groups are not large. However, COPD patients believe that the disease will progressively deteriorate, see more physical limitations, **but experience less pain** and are of the opinion that their disease can be better controlled by medical treatment than people with other chronic conditions (Patiëntenpanel, 2000).” p. 124	Symptom of COPD
		In section Quality of Life in Patients with COPD: Psychological Well-Being, Other Considerations,<br>“If the patient experiences dyspnoea, the doctor should ask to what extent this leads to feelings of anxiety and worry (real or unreal). Also ask under which circumstances this is most common (ie physical exertion). Accompanying symptoms that the doctor asks about or in which he should think of the existence of an anxiety disorder are: palpitations, perspiration, tremor, **pain,** upset stomach, tingling, deaf feelings, heat or cold sensations, de-realization or depersonalization feelings, restlessness, being quickly tired, concentration problems, irritability, sleep problems. Depending on the severity, duration and course of the complaints, the influence	Physician visit to assess for differential diagnosis (anxiety)

on social functioning, and avoidance of certain situations or activities, the doctor decides whether targeted treatment is necessary.” p. 129	
In section Diagnosis for Lung Rehabilitation, Screening, Other considerations,<br>“One problem with the screening lists is that lung patients often report **thoracic pain**. A complication is that exercise tests are not only time consuming, But also require expertise and medical supervision. Standard ergometric screening therefore rely heavily on capacity. For lung patients, (risk) groups should be defined based on the severity of the disorder (effort, desaturation), cardiovascular co-morbidity and training intensity.” p. 190	Physician visit to assess for pulmonary rehabilitation
In Table 17.2 Most important nuisance side effects of nicotine replacement drugs,<br>“Chewing gum: Hiccups, gastrointestinal complaints, **jaw pain**, mouth–tooth complaints.” p. 273	Drug adverse effects (nicotine replacement therapy)
In section Bupropion,<br>“Side effects: Very common (>10%): insomnia. Common (1–10%): dry mouth, headache, dizziness, tremor, agitation, anxiety disorders, depression, difficulty concentrating, gastrointestinal disorders (such as nausea, vomiting, **abdominal pain** and constipation), taste disturbances, fever, perspiration, acute exanthema, itching, urticaria. Uncommon (0.1–1%): tachycardia, increased blood pressure (sometimes severe), flushing, **chest pain**, asthenia, anorexia, confusion, tinnitus, visual disturbances.” p. 316	Drug adverse effects (smoking cessation aid—Bupropion)
In section Varenicline,<br>“Side effects. Uncommon (0.1–1%): upper respiratory tract infections, throat irritation, hoarseness, mycosis, loss of appetite, anorexia, polydipsia, panic attack, bradyphrenia, abnormal thinking, mood swings, tremor, coordination disorders, dysarthria, hypertonia, restlessness, hypoaesthesia, hypoglyphosis , lethargy, asthenia, altered libido, menorrhagia, vaginal discharge, sexual dysfunction, cardiac arrhythmia, palpitations, visual disturbances, sclera discoloration, **eye pain**, increased tear production, photophobia, oral mucosal diseases, altered intestinal tract, **abdominal pain**, gastritis, gastroesophageal reflux, regurgitation, skin rash, itching, acne, increased perspiration, stiff joints, muscle spasms, costochondritis, snoring,	Drug adverse effects (Nicotine replacement therapies—Varenicline)

		sleep disorders, malaise, **pain**, fever, hypertension, tachycardia, weight gain, ecg abnormalities, abnormal sperm, glucosuria, nocturia, polyuria, abnormal liver function tests, reduced platelet count, decreased serum calcium concentration, increased CRP." p. 318–319	
Malaysia [46] **2009**	1	In section COPD and Surgery, Recommendations: Surgery in COPD Patients<br>"The following measures help minimise pulmonary complications in at-risk patients:<br>Postoperative<br>• Adequate **pain control**<br>- consider epidural analgesia in at-risk patients<br>- avoid opiates…." p. 32	Pharmacological management (post-surgery)
CTS: Canada [48,49] **2007/8**	1	In Table 7 Pharmacological aids to smoking cessation column,<br>"Adverse effects: Burning, **jaw pain**, hiccups." p. 13	Drug adverse effects (Nicorette gum)
Germany [50] **2007**	2	In section Palliative Care,<br>"Dyspnoea, fatigue, depression and **pain** are the most common symptom in patients in the year before death." p. 24	Symptom of COPD
		In section Bronchodilators—Theophylline,<br>"Significant undesirable effects of Theophylline therapy are nausea, vomiting**, abdominal pain,** sleep disorders, muscle cramps, hypokalaemia and tachycardic heart rhythm disturbances, which occasionally already at serum concentrations in the therapeutic range." p. 41	Drug adverse effects (methylxanthines)

References

1. From the Global Strategy for the Diagnosis, Management and Prevention of COPD Global Initiative for Chronic Obstructive Lung Disease (GOLD) 2019. Available online: http://www.goldcopd.org/ (accessed on 12 November 2018).
2. The Lung Foundation Australia The COPD-X plan Australian and New Zealand guidelines for the management of chronic obstructive pulmonary disease 2018, version 2.55, August 2018. Available online: http://copdx.org.au/copd-x-plan/ (accessed on 17 December 2018).
3. Vogelmeier, C.; Buhl, R.; Burghuber, O.C.; Criee, C.P.; Ewig, S.; Godnic-Cvar, J.; Hartl, S.; Herth, F.; Kardos, P.; Kenn, K.; et al. Guideline for the Diagnosis and Treatment of COPD Patients Issued by the German Respiratory Society and the German Atemwegsliga in Cooperation with the Austrian Society of Pneumology. *Pneumologie* **2018**, *72*, 253–308, [PubMed].
4. Stolz, D.; Barandun, J.; Borer, H.; Bridevaux, P.O.; Brun, P.; Brutsche, M.; Clarenbach, C.; Eich, C.; Fiechter., Frey, M.; et al. Diagnosis, Prevention and Treatment of Stable COPD and Acute Exacerbations of COPD: The Swiss Recommendations. *Respiration* **2018**, *96*, 382–398, [PubMed].
5. Park, Y.B.; Rhee, C.K.; Yoon, H.K.; Oh, Y.M.; Lim, S.Y.; Lee, J.H.; Yoo, K.H.; Ahn, A.; Committee of the Korean COPD Guideline 2018. Revised (2018) COPD Clinical Practice Guideline of the Korean Academy of Tuberculosis and Respiratory Disease: A Summary. *Tuberc Respir Dis (Seoul)* **2018**, 81, 261–273, [PubMed].
6. National Board of Health and Welfare Socialstyrelsen [National guidelines for care for asthma and COPD - Support for management]. Available on: http://www.socialstyrelsen.se/Lists/Artikelkatalog/Attachments/20858/2018-1-36.pdf (accessed on 5 December 2018). .
7. Ministry of Health British Columbia Chronic obstructive pulmonary disease (COPD): Diagnosis and Management. Available online: https://bit.ly/2AA2spd (accessed on 29 November 2018).
8. Mahboub, B.H.; Vats, M.G.; Al Zaabi, A.; Iqbal, M.N.; Safwat, T.; Al-Hurish, F.; Miravitlles, M.; Singh, D.; Asad, K.; Zeineldine, S.; et al. Joint statement for the diagnosis, management, and prevention of chronic obstructive pulmonary disease for Gulf Cooperation Council countries and Middle East-North Africa region, 2017. *Int J Chron Obstruct Pulmon Dis* **2017**, *12*, 2869–2890, [PubMed].
9. University of Michigan Health System Chronic obstructive pulmonary disease. Available online: www.uofmhealth.org/provider/clinical-care-guidelines (accessed on 29 November 2018).
10. Lim ,T.K.; Chee, C.B.; Chow, P.; Chua, G.S.W.; Eng, S.K.; Goh, S.K.; Kng, K.K.; Lim, W.H.; Ng, T.P.; Ong, T.H.; et al. Ministry of Health Clinical Practice Guidelines: Chronic Obstructive Pulmonary Disease. *Singapore Medical Journal* **2018**, 59, 76-86.
11. From the Global Strategy for the Diagnosis, Management and Prevention of COPD. Global Initiative for Chronic Obstructive Lung Disease (GOLD) 2015. Available online: http://www.goldcopd.org/ (accessed on 27 April 2016).
12. The Lung Foundation The COPD-X plan Australian and New Zealand guidelines for the management of chronic obstructive pulmonary disease 2017, version 2.49, March 2017 Available online: http://copdx.org.au/copd-x-plan/ (accessed on 1 April 2017).
13. Institute for Clinical Systems Improvement Diagnosis and management of chronic obstructive pulmonary disease (COPD). Available on: https://www.icsi.org/guidelines__more/catalog_guidelines_and_more/catalog_guidelines/catalog_respiratory_guidelines/copd/ (accessed on 27 April 2016).
14. National Board of Health and Welfare Socialstyrelsen [National guidelines for care for asthma and COPD - Support for management]. Available online: http://www.socialstyrelsen.se/Lists/Artikelkatalog/Attachments/20858/2018-1-36.pdf (accessed on 5 December 2018).
15. NHG Working Group on Asthma and COPD in adults. [NHG standard COPD (Third revision)]. *Huisarts Wet* **2015**, *58*, 198-211.

16. Khan, J.H.; Lababidi, H.M.; Al-Moamary, M.S.; Zeitouni, M.O.; AL-Jahdali, H.H.; Al-Amoudi, O.S.; Wali, S.O.; Idrees, M.M.; Al-Shimemri, A.A.; Al Ghobain, M.O.; et al. The Saudi guidelines for the diagnosis and management of COPD. *Ann Thorac Med* **2014**; *9*, 55–76, [PubMed].
17. Gupta, D.; Agarwal, R.; Aggarwal, A.N.; Maturu, V.N.; Dhooria, S.; Prasad, K.T.; Sehgal, I.S.; Yenge, L.B.; Jindal, A.; Singh, N.; et al. Guidelines for diagnosis and management of chronic obstructive pulmonary disease: Joint ICS/NCCP (I) recommendations. *Lung India* **2013**, *30*, 228–267, [PubMed].
18. Bettoncelli, G.; Blasi, F.; Brusasco, V.; Centanni, S.; Corrado, A.; De Benedetto, F.; De Michele, F.; Di Maria, G.U.; Donner, C.F.; Falcone, F.; et al. The clinical and integrated management of COPD. An official document of AIMAR (Interdisciplinary Association for Research in Lung Disease), AIPO (Italian Association of Hospital Pulmonologists), IMER (Italian Society of Respiratory Medicine), SIMG (Italian Society of General Medicine). *Multidiscip Respir Med* **2014**, *9*, doi: 10.1186/2049-6958-9-25.
19. Department of Veterans Affairs, Department of Defence VA/DoD clinical practice guideline for the management of chronic obstructive pulmonary disease 2014. Available online: http://www.healthquality.va.gov/guidelines/cd/copd/ (accessed on 27 April 2016).
20. Chuchalin A.G.; Avdeev, S.N.; Aysanov, Z.R.; Belevskiy, A.S.; Leshchenko, I.V.; Meshcheryakova, N.N., Ovcharenko, S.I.; Shmelevet, E.I. Russian Respiratory Society Federal guidelines on diagnosis and treatment of chronic obstructive pulmonary disease. *Russian Pulmonology* **2014**, *3*, doi: 10.18093/0869-0189-2014-0-3-15-54.
21. Kankaanranta, H.; Harju, T.; Kilpelainen, M.; Mazur, W.; Lehto, J.T.; Katajisto, M.; Peisa, T.; Meinander, T.; Lehtimäki, L. Diagnosis and pharmacotherapy of stable chronic obstructive pulmonary disease: The Finnish guidelines. *Basic Clin Pharmacol Toxicol*. **2015**, *116*, 291–307, [PubMed].
22. Sliwinski, P.; Gorecka, D.; Jassem, E.; Pierzchala, W. [Polish Respiratory Society guidelines for chronic obstructive pulmonary disease]. *Pneumonologia i alergologia polska* **2014**, *82*, 227–263.
23. High Authority of Health [Care initiative guide: chronic obstructive pulmonary disease]. Available on: http://www.has-sante.fr/portail/upload/docs/application/pdf/2012-04/guide_parcours_de_soins_bpco_finale.pdf (accessed on 27 April 2016). .
24. Kocabaş, A.; Atış, S.; Çöplü, L.; Erdinç, E.; Ergan, B.; Gürgün, A.; Koktürk, N.; Polatlı, M.; Şen, E.; Yıldırım, N. Kronik Obstrüktif Akciğer Hastaliği (KOAH) Koruma, Tani ve Tedavi Raporu 2014, *Turkish Thoracic Journal* **2014**. S1–S76 [https://www.chiesi.com.tr/img/download/documenti/363_ttd-koah-tani-ve-tedav-C4-B0-raporu-2014.pdf].
25. Korea Foundation for Tuberculosis and Respiratory Disease COPD 진료지침 2014 개정. Available online: http://www.kaim.or.kr/files/guide/호흡기_02.pdf (accessed on 29 November 2018).
26. Montes de Oca, M.; Varela, M.V.L.; Acuña, A.; Schiavi, E.; Rey, M.A.; Jardim, J.; Casas, A.; Tokumoto, A.; Torres Duque, C.A.; Ramírez-Venegas, A.; et al. [Latinoamericana COPD guide - 2014 Based on evidence]. *Arch Bronconeumol* **2015**, 3–43, doi.org/10.1016/j.arbres.2014.11.017.
27. Miravitlles, M.; Soler-Cataluña, J.J.; Calle, M.; Molina, J.; Almagro, P.; Quintano, J.A.; Riesco, J.A., Triqueros, J.A., Piñera, P.; Simón A.; et al. Spanish guideline for COPD (GesEPOC). Update 2014. *Arch Bronconeumol* **2014**, *50*, doi: 10.1016/S0300-2896(14)70070-5.
28. Task Force of GesEPOC. Clinical practice guideline for the diagnosis and treatment of patients with chronic obstructive pulmonary disease (COPD) – Spanish guideline for COPD (GesEPOC). *Arch Bronconeumol* **2012,** *48*, doi: 10.1016/S0300-2896(12)70035-2.
29. Koblizek, V.; Chlumsky, J.; Zindr, V.; Neumannova, K.; Zatloukal, J.; Zak, J.; Sedlak, V.; Kocianova, J.; Zatloukal, J.; Hejduk, K.; et al. Chronic obstructive pulmonary disease: Official diagnosis and treatment guidelines of the Czech Pneumological and Phthisiological Society; a novel phenotypic approach to COPD with patient-oriented care. *Biomed PAP* **2013**, *157*, 189–201, doi: 10.5507/bp.2013.039.
30. Russi, E.; Karrer, W.; Brutsche, M.; Eich, C.; Fitting, J.; Frey, M.; Geiser, T.; Kuhn, M.; Nicod, L.; Quadri, F.; et al. Diagnosis and management of chronic obstructive pulmonary disease: The Swiss guidelines. *Respiration* **2013**, *85*, 160–174, [PubMed].

31. Chinese Medical Association Respiratory Diseases Society Chronic Obstructive Pulmonary Diseases Group. Guidelines for the diagnosis and treatment of chronic obstructive pulmonary disease (revised edition 2013). *Chinese J Front Med* **2014**, *6*, 67–80.
32. Ministry of Health Chile [Clinical guide chronic obstructive pulmonary disease COPD]. Available on: http://www. supersalud.gob.cl/difusion/572/articles-655_recurso_ 1.pdf (accessed on 27 April 2016).
33. Ministry of Health Ukraine [Chronic obstructive pul- monary disease adapted clinical guidelines based on evidence]. Available on: http://www.dec.gov.ua/mtd/ hozl.html accessed on 29 April 2016).
34. Department of Quality in Health [Diagnosis and Treatment of Chronic Obstructive Pulmonary Disease]. Available on: https://www.dgs.pt/directrizes-da-dgs/normas-e-circulares-normativas/norma-n-0282011-de-30092011-atualizada-a-10092013-png.aspx (accessed on 29 November 2018). .
35. Norwegian Directorate of Health [COPD national academic policy and guidelines for prevention, diagnosis and care]. Available on: https://helsedirektoratet.no/Lists/Publikasjoner/Attachments/847/Nasjonal-faglig-retningslinje-og-veileder-for-forebygging-diagnostisering-og-oppfølging-IS-2029.pdf (accessed on 29 November 2018).
36. University of Michigan Health System Chronic obstructive pulmonary disease. Available on: http://www.med.umich.edu/1info/FHP/practiceguides/copd/copd.pdf (accessed on 27 April 2016).
37. Danish Society of Respiratory Medicine Danske KOL-Guidelines [Danish COPD guidelines]. Available on: https://www.lungemedicin.dk/fagligt/101-dansk-kol-retningslinje-2012/file.html (accessed on 29 November 2018). .
38. Figueroa Casas, J.C.; Schiavi, E.; Mazzei, J.A.; Lopez, A.M.; Rhodius, E.; Ciruzzi, J.; Sívori, M.; Grupo Recomendaciones De EPOC; Asociacion Argentina De Medicina Respiratoria. [Recommendations for the prevention, diagnosis and treatment of COPD in Argentina]. *Medicina (B Aires)* **2012**, *72*, Supplement 1, 1-33, [PubMed].
39. Qaseem, A.; Wilt, T.J.; Weinberger, S.E.; Hanania, N.A. Criner, G.; Van der Molen, T.; Marciniuk, D.D.; Denberg, T.; Schünemann, H.; Wedzicha, W.; et al. Diagnosis and management of stable chronic obstructive pulmonary disease: A clinical practice guideline update from the American College of Physicians, American College of Chest Physicians, American Thoracic Society, and European Respiratory Society. *Ann Intern Med* **2011**, *155*, 179–191, [PubMed].
40. Abdool-Gaffar, M.; Ambaram, A.; Ainslie, G.; Bolliger, C.; Feldman, C.; Geffen, L.; Irusen, E.M.; Joubert, J.; Lalloo, U.G.; Mabaso, T.T.; et al. Guideline for the management of chronic obstructive pulmonary disease: 2011 update. *S Afr Med J* **2011**, *101*, 63–73, [PubMed].
41. Guidelines and Protocols Advisory Committee Chronic obstructive pulmonary disease (COPD). Available on: http://www2.gov.bc.ca/assets/gov/health/practitioner-pro/bc-guide lines/copd.pdf (accessed on 27 April 2016).
42. Huizar-Hernández, V.; Rodríguez-Parga, D.; Sánchez-Mécatl, M.Á.; Nájera-Cruz, M.P.; Alvarado-Fuentes, J.; del Pilar Torres-Arreola, L. [Clinical practice guideline diagnosis and treatment of chronic obstructive lung disease]. *Rev Med Inst Mex Seguro Soc* **2011**, 49, 89–100, [PubMed].
43. National Institute for Health and Care Excellence Chronic obstructive pulmonary disease: Management of chronic obstructive pulmonary disease in adults in primary and secondary care (update guideline). Available online: https://www.nice.org.uk/guidance/conditions-and-diseases/respiratory-conditions/chronic-obstructive-pulmonary-disease (accessed on 13 April 2016).
44. The Institute for Healthcare Improvement CBO [Guideline for the diagnosis and treatment of COPD update March 2010]. Available on: http://www.nvalt.nl/uploads/Mn/U9/MnU99pqRBYK7a_zStHhumA/Richtlijn-Diagnostiek-en-behandeling-van-COPD-maart-2010.pdf (accessed on 27 April 2016).
45. French Language Pneumology Society. [Recommendation for clinical practice management of COPD]. *Rev Mal Respir* **2010**, *27*: 522–548.

46. Ministry of Health Malaysia, Academy of Medicine Malaysia, Malaysian Thoracic Society Management of chronic obstructive pulmonary disease 2009. Available online: http:// www.moh.gov.my/attachments/4749.pdf (accessed on 29 April 2016).
47. Report of the Council on COPD and Pulmonary Rehabilitation Philippine College of Chest Physicians Clinical practice guidelines in the diagnosis and management of chronic obstructive pulmonary disease (COPD) in the Philippines. Available online: http://philchest.org/v3/wp-content/uploads/2013/05/PHILIPPINES-COPD-CPGuidelines-2009.pdf (accessed on 29 November 2018).
48. O'Donnell, D.E.; Aaron, S.; Bourbeau, J.; Hernandez, P.; Marciniuk, D.D.; Balter, M.; Ford, G.; Gervais, A.; Goldstein, R.; Hodder, R.; et al. Canadian Thoracic Society recommendations for management of chronic obstructive pulmonary disease–2007 update. *Can Respir J* **2007**, 5B–32B, doi: 10.1155/2007/830570, [PubMed].
49. O'Donnell, D.E.; Hernandez, P.; Kaplan, A.; Aaron, S.; Bourbeau, J.; Marciniuk, D.; Balter, M.; Ford, G. Gervais, A.; Lacasse, Y.; et al. Canadian Thoracic Society recommendations for management of chronic obstructive pulmonary disease - 2008 update - highlights for primary care. *Can Respir J* **2008**, *15*, 1A–8A, doi: 10.1155/2008/641965, [PubMed].
50. Vogelmeier, C.; Buhl, R.; Criee, C.P.; Gillissen, A.; Kardos, P.; Kohler, D.; Magnussen, H.; Morr, H.; Nowak, D.; Pfeiffer-Kascha, D.; et al. Guidelines for the diagnosis and therapy of COPD issued by Deutsche Atemwegsliga and Deutsche Gesellschaft fur Pneumologie und Beatmungsmedizin. *Pneumologie*. **2007**, *61*, e1–40, [PubMed].
51. Bellamy, D.; Bouchard, J.; Henrichsen, S.; Johansson, G.; Langhammer, A.; Reid, J.; van Weel, C.; Buist, S.; International Primary Care Respiratory Group (IPCRG) Guidelines: management of chronic obstructive pulmonary disease (COPD). *Prim Care Respir J* **2006**, 15, 48–57, [PubMed].
52. Doherty, D.E.; Belfer, M.H.; Brunton, S.; Fromer, L.; Morris, C.; Snader, T. Chronic obstructive pulmonary disease: Consensus recommendations for early diagnosis and treatment. *J Fam Pract*. **2006**, *55*, S1–8, [http://www.pcrg-us.org/UserFiles/COPD%20JFP.pdf].
